# Development and external validation of a machine learning model based on multimodal dynamic data for predicting shunt-dependent hydrocephalus after aneurysmal subarachnoid hemorrhage: a dual-center retrospective cohort study

**DOI:** 10.3389/fneur.2026.1837898

**Published:** 2026-07-24

**Authors:** Chengzhang Zheng, Jianhuang Huang, Xinguo Wang, Qing Ye, Caihou Lin, Xiang Gu

**Affiliations:** 1 Department of Neurosurgery, Mindong Hospital of Ningde City, Ningde, Fujian, China; 2 Department of Neurosurgery, Affiliated Hospital of Putian University, Putian, Fujian, China; 3 Department of Neurosurgery, Fujian Medical University Union Hospital, Fuzhou, Fujian, China

**Keywords:** aneurysmal subarachnoid hemorrhage, external validation, machine learning, random forest, risk prediction, shunt-dependent hydrocephalus

## Abstract

**Objective:**

To develop and externally validate a machine learning model for predicting shunt-dependent hydrocephalus (SDHC) after aneurysmal subarachnoid hemorrhage (aSAH) using multimodal dynamic data.

**Methods:**

This dual-center retrospective study enrolled aSAH patients from Mindong Hospital (development cohort, 2018–2025) and Putian University Hospital (external validation cohort, 2016–2025). Using a day-21 landmark design, static baseline variables, weekly dynamic parameters, and clinical interventions were collected. Derived features capturing ventricular dynamics, intracranial pressure fluctuations, and inflammatory trajectories were engineered. Three models were compared using nested cross-validation. Performance was evaluated by AUC, AUPRC, Brier score, decision curve analysis, and SHAP analysis. Derived dynamic features characterizing ventricular expansion, intracranial pressure fluctuation, and inflammatory trajectories were engineered, and an ablation analysis was performed to quantify their incremental predictive contribution beyond static baseline variables.

**Results:**

The development cohort included 228 patients (SDHC 21.9%); external validation included 102 patients (SDHC 23.5%). Random forest achieved optimal internal validation performance (AUC 0.894, AUPRC 0.676, Brier 0.112). At threshold 0.30, sensitivity and specificity were 0.920 and 0.840. External validation showed robust generalizability (AUC 0.867, sensitivity 0.875, specificity 0.859). Risk stratification demonstrated SDHC rates of 5.3, 35.7, and 64.7% in low-, intermediate-, and high-risk groups (*p* < 0.001). Key predictors included GCS score, Hunt-Hess grade, Evans index change, age, ICP variability, and 14-day CSF drainage volume.

**Conclusion:**

The random forest model integrating multimodal dynamic data accurately predicts SDHC after aSAH with strong external validity; ablation analysis confirmed that incorporating dynamic features significantly improved discrimination (AUC 0.894 vs. 0.831 for the static-only model, ΔAUC = 0.063, *p* = 0.028), identifying dynamic monitoring data — rather than algorithm choice alone — as a key driver of predictive performance and facilitating early identification and risk stratification of high-risk patients.

## 1 Introduction

Aneurysmal subarachnoid hemorrhage (aSAH) is a devastating cerebrovascular emergency associated with high mortality and morbidity (1). Although advances in early diagnosis and treatment strategies for intracranial aneurysms have been achieved in recent years, post-aSAH complications continue to exert a significant adverse impact on long-term patient prognosis. Among these complications, shunt-dependent hydrocephalus (SDHC) represents one of the most consequential sequelae (2). Prior studies have reported an incidence of SDHC ranging from approximately 15.0 to 28.8% among aSAH patients (3–5). Once SDHC develops, patients typically require permanent ventriculoperitoneal shunt (VPS) implantation, which not only prolongs hospitalization and consumes substantial healthcare resources but may also further compromise quality of life and neurological recovery through device-related complications such as infection, obstruction, and overdrainage (6, 7).

In clinical practice, tools such as the Hunt-Hess grade and modified Fisher scale are commonly used to assess aSAH severity and associated complication risk (4, 8). However, existing evidence on SDHC prediction reveals three major, interrelated gaps. First, traditional scoring systems rely primarily on static indicators obtained at admission or from early imaging, and their predictive performance for SDHC is limited, with reported AUC values predominantly in the range of 0.60–0.70 (6, 9, 10). Second, although several studies have integrated imaging, cerebrospinal fluid, and serological parameters into predictive scores such as CHESS, SDASH, and MAI, with promising results (11–13), these models still rely on variables measured at one or a few time points and rarely incorporate continuous in-hospital time-series monitoring; consequently, the independent, quantifiable contribution of dynamic physiological trajectories — progressive ventricular dilation, ICP fluctuation, inflammatory evolution, and cumulative CSF drainage — to predictive performance has not been rigorously established (2, 14). Third, most existing predictive models, including machine learning-based ones, have been developed and validated within a single center, without independent multi-center external validation or model interpretability analysis, limiting confidence in their generalizability and clinical acceptance (6, 13). These three gaps directly motivate the three objectives of the present study: (1) to construct a clinically interpretable dynamic feature set from multimodal time-series data and quantify its incremental predictive value through ablation analysis; (2) to perform a horizontal comparison of three representative machine learning paradigms (logistic regression, random forest, and XGBoost); and (3) to complete dual-center external validation together with SHAP-based interpretability analysis. The selection of day 21 after aSAH onset as the prediction landmark is grounded in the underlying pathophysiology of SDHC. During the first three weeks after hemorrhage, breakdown products of subarachnoid and intraventricular blood trigger arachnoid granulation fibrosis and impaired CSF reabsorption, which manifest clinically and radiologically as progressive ventricular dilation, unstable intracranial pressure, and a sustained inflammatory response (2, 14). These processes evolve gradually rather than being fully expressed within the first days after ictus, so that a single early assessment captures only a snapshot of an ongoing trajectory. A three-week window allows these dynamic processes to be captured with adequate temporal resolution while remaining compatible with routine weekly clinical monitoring schedules, providing both a mechanistic and a pragmatic basis for the landmark design adopted in this study. In China, no unified dynamic risk-stratification tool is currently available across neurosurgical departments of varying resource levels; decisions regarding the timing of shunt evaluation and drainage management therefore rely largely on individual clinicians’ experience, underscoring the practical need for a validated, data-driven prediction tool applicable across different tiers of care.

In recent years, machine learning techniques have provided new methodological tools for integrating complex multimodal clinical data and identifying latent predictive patterns that may be missed by conventional statistical methods. Unlike traditional logistic regression, which assumes linear relationships between predictors and outcomes, machine learning algorithms such as random forest and gradient-boosted trees can automatically capture nonlinear relationships and high-order interactions among variables, making them particularly suited for modeling the complex, multifactorial pathogenesis of SDHC. Emerging evidence suggests that, compared with conventional statistical models, machine learning approaches may offer superior predictive performance for SDHC after aSAH, with reported AUC values exceeding 0.80 (6). Nevertheless, most existing machine learning studies have focused predominantly on baseline features, with a notable lack of systematic integration and in-depth exploitation of dynamic temporal information collected during hospitalization. Against this background, the present study aims to conduct a dual-center retrospective cohort study in which static baseline characteristics, in-hospital dynamic monitoring parameters, and key clinical intervention events are systematically collected. Through derived feature engineering, clinically interpretable dynamic predictive variables are constructed. On this basis, three representative machine learning algorithms—logistic regression (as a classical benchmark), random forest (bagging-based ensemble learning), and XGBoost (boosting-based ensemble learning)—covering the major algorithmic paradigms for structured clinical data, are compared. A dynamic model for predicting SDHC risk is developed and externally validated, with the goal of providing more precise decision support for the early identification and subsequent management of high-risk patients.

## 2 Methods

### 2.1 Study design and data sources

**Figure 1:**
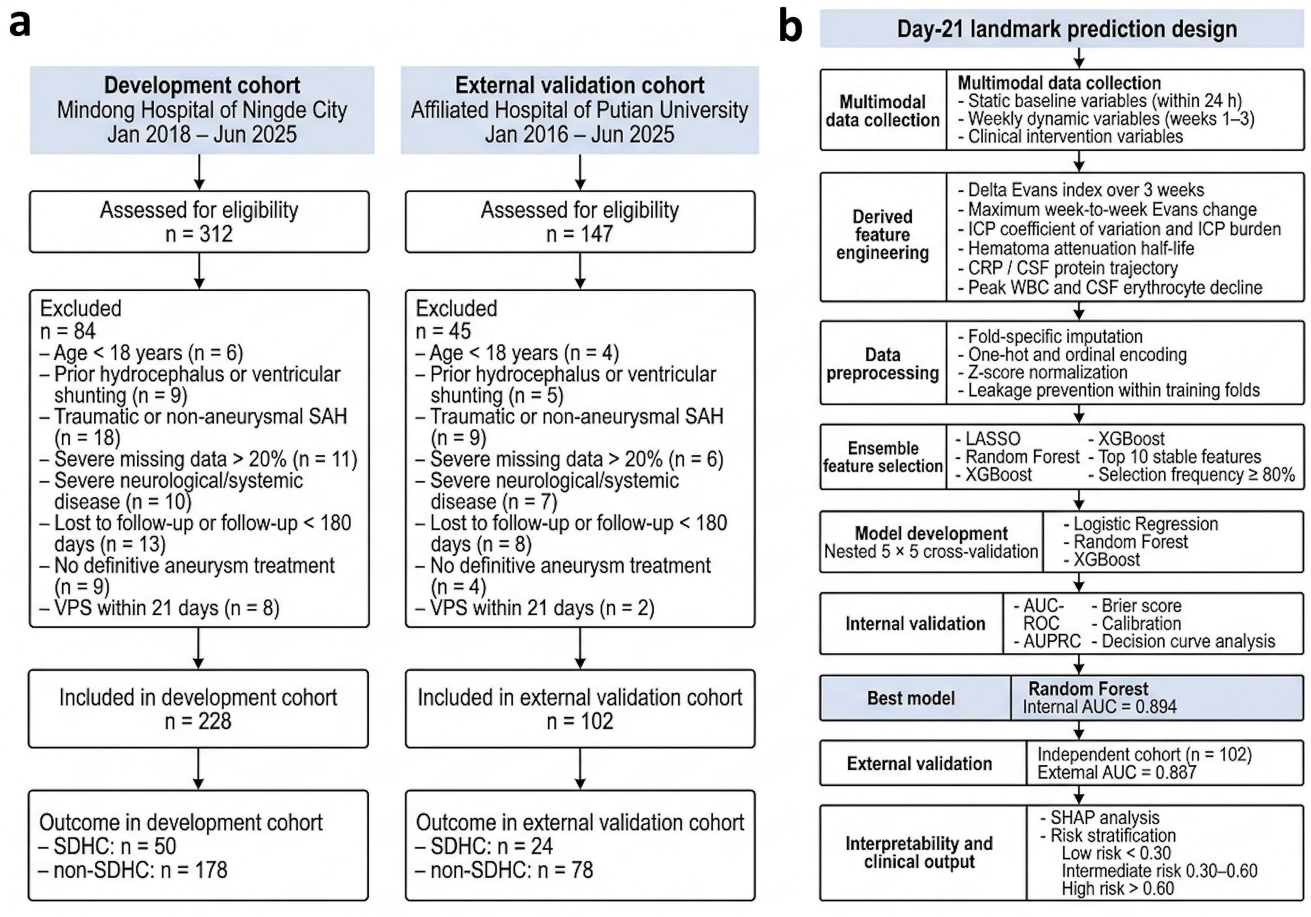
Study workflow and technical roadmap. **(a)** Patient screening and cohort construction for the development cohort and the external validation cohort. **(b)** Workflow of model development and validation, including day-21 landmark design, multimodal data collection, derived feature engineering, preprocessing, ensemble feature selection, nested cross-validation, external validation, and SHAP-based interpretability analysis.

This was a dual-center retrospective cohort study comprising a model development cohort and an independent external validation cohort. The study design, data processing, and model development workflow are presented in Figure 1.

#### 2.1.1 Model development cohort

The study population consisted of patients with aSAH admitted to the Department of Neurosurgery, Mindong Hospital of Ningde City, between January 2018 and June 2025. All patients received definitive aneurysm treatment (endovascular coil embolization or microsurgical clipping) and completed a minimum 180-day follow-up. The study was approved by the Medical Research Ethics Committee of Mindong Hospital of Ningde City (approval number: K2026020901); informed consent was waived given the retrospective study design.

#### 2.1.2 External validation cohort

To assess the generalizability and external validity of the model, patients with aSAH admitted to the Department of Neurosurgery, Affiliated Hospital of Putian University, between January 2016 and June 2025 were enrolled as an independent external validation set. The same inclusion and exclusion criteria, variable definitions, and data collection procedures as those used in the development cohort were applied at this center. The study was approved by the Medical Research Ethics Committee of the Affiliated Hospital of Putian University (approval number: 202555), with a waiver of patient informed consent.

### 2.2 Inclusion and exclusion criteria

Patients were required to meet the following criteria (2, 10, 15). *Inclusion criteria*: (1) aSAH confirmed by CTA or DSA, with identification of a causative intracranial aneurysm; (2) age ≥18 years; (3) underwent surgical treatment for the ruptured aneurysm (microsurgical clipping or endovascular coil embolization); (4) completion of at least 180 days of clinical follow-up. *Exclusion criteria*: (1) prior history of hydrocephalus or ventricular shunting; (2) SAH of traumatic or non-aneurysmal etiology; (3) severe missing clinical data (>20% of key variables); (4) concomitant severe neurological conditions (e.g., severe traumatic brain injury, brain tumor) or systemic diseases (end-stage hepatic or renal failure) expected to significantly affect prognosis; (5) VPS implantation within 21 days of admission; (6) loss to follow-up or follow-up duration <180 days. Patients undergoing VPS implantation within 21 days of admission were excluded because early shunt dependence typically reflects acute, refractory hydrocephalus driven by mechanisms distinct from the gradual, arachnoid fibrosis-mediated process targeted by this landmark model (Section 2.3.1). This exclusion defines the intended applicable population of the model and does not imply that early-onset hydrocephalus is being modeled or that the tool is validated for that distinct clinical scenario.

### 2.3 Variable definitions and data collection

#### 2.3.1 Outcome variable

The primary outcome variable was SDHC, defined as permanent VPS implantation performed for communicating hydrocephalus within 180 days following aSAH onset (4). Given that this study employed a day-21 landmark prediction strategy to ensure temporal consistency, the outcome events for model prediction were restricted to VPS procedures occurring after day 21 and within 180 days of aSAH onset. Accordingly, only patients who remained in the at-risk set at day 21 and had not previously undergone VPS were included in the predictive analysis.

VPS implantation required simultaneous fulfillment of two core criteria: (1) clinical criteria—worsening consciousness, gait disturbance, urinary incontinence, or cognitive decline that persisted during hospitalization or after discharge, with other causes (e.g., re-bleeding) excluded; and (2) radiological criteria—progressive ventricular enlargement on follow-up CT or MRI (Evans index >0.30 or an increase of >10% from baseline) without mechanical obstruction of CSF pathways. In addition to the above two core criteria, a positive CSF tap test could serve as supporting evidence for the VPS decision. All outcome data were verified through electronic medical records and follow-up documentation; in both centers, two independent neurosurgeons adjudicated outcomes, and discrepancies were resolved through discussion and consensus. Identical outcome definitions were applied to both the development and external validation cohorts.

We acknowledge that the definition of SDHC varies across the literature. Some centers define SDHC based on failure of EVD weaning trials or the persistence of clinical and radiological signs of hydrocephalus, irrespective of the time point (2, 12, 16, 17). The present study adopted the commonly used criterion of VPS implantation within 180 days, consistent with multiple prior studies (4, 14). However, this definition is inherently influenced by clinical decision-making and institutional practices. In our cohorts, only 5 patients (2.1%) in the development cohort underwent VPS before day 21, suggesting that early SDHC was uncommon in this population. Future studies using standardized, process-based definitions (e.g., structured EVD weaning protocols) may provide more precise outcome ascertainment.

#### 2.3.2 Predictor variables

The following three categories of predictor variables were systematically collected:

##### 2.3.2.1 Static baseline variables (within 24 h of admission)

These included demographic information (age, sex), medical history (hypertension, diabetes mellitus, etc.), personal history (smoking, alcohol use), disease severity at admission (Glasgow Coma Scale [GCS] score, Hunt-Hess grade, modified Fisher grade), aneurysm characteristics (location, maximum diameter), treatment modality (surgical clipping or endovascular intervention), and timing of intervention (4, 10, 14, 18, 19).

##### 2.3.2.2 Dynamic time-series variables

Head CT scans were performed according to institutional protocols at both centers: routinely at admission, within 24 h postoperatively, and subsequently at intervals of approximately 5–7 days during hospitalization or as clinically indicated (e.g., neurological deterioration). On average, each patient underwent 4–6 CT scans during the three-week observation period, providing sufficient imaging data for weekly aggregation of CT-based variables including Evans index and cistern CT attenuation values.

These variables covered the first three weeks of hospitalization and were aggregated on a weekly basis. They included intracranial pressure (ICP) measured by lumbar puncture; weekly mean ICP, weekly Evans index (defined as the ratio of the maximum width of the frontal horns of the lateral ventricles to the maximum internal diameter of the skull at the same level, measured on axial CT), CT attenuation values of hematoma/edema in the ambient and basal cisterns, weekly white blood cell (WBC) count, neutrophil ratio, C-reactive protein (CRP), procalcitonin (PCT), CSF protein, CSF red and white cell counts, serum sodium, and blood glucose (2, 20, 21). Evans index was measured on a fixed soft-tissue window (window width 80 HU, window level 35 HU) on the institutional PACS workstation, at the axial slice showing the maximal frontal horn width at the level of the foramen of Monro. Each measurement was performed by one trained investigator and verified by a second investigator, blinded to outcome status; discrepancies were resolved by consensus. An intracranial pressure value >200 mmH2O was considered elevated. Cumulative CSF drainage volume was defined as the total (running-sum) drainage volume accumulated from initiation of drainage through postoperative day 3 and through postoperative day 14, respectively, rather than a daily average.

##### 2.3.2.3 Clinical intervention variables

These included delayed cerebral ischemia (DCI), re-bleeding, lumbar drain placement (22), cumulative CSF drainage volume (at postoperative days 3 and 14), and central nervous system infection (10, 14).

### 2.4 Derived feature engineering

To characterize the dynamic pathophysiological changes underlying SDHC development, several clinically interpretable derived features were constructed from the raw time-series data: (1) Ventricular expansion dynamics: The Evans index was measured on axial non-contrast CT at the level of the foramen of Monro, defined as the ratio of the maximum width of the frontal horns of the lateral ventricles to the maximum internal diameter of the skull at the same level. Evans index measurements were obtained at weeks 1, 2, and 3 during hospitalization by trained investigators using standardized measurement protocols, with measurements reviewed by a second investigator. The total change in Evans index (ΔEvans_total) was calculated as ΔEvans_total = Evans_week3 − Evans_week1. For example, if a patient’s Evans index was 0.25 at week 1 and 0.30 at week 3, ΔEvans_total = +0.05, indicating progressive ventricular dilation. Additionally, the maximum week-to-week absolute change in Evans index (|ΔEvans_max|) was calculated as max(|Evans_week2 − Evans_week1|, |Evans_week3 − Evans_week2|). These derived metrics quantify the temporal trajectory of ventricular size change, with positive ΔEvans_total values reflecting progressive enlargement consistent with evolving hydrocephalus (23). The Evans index was measured on axial CT at the level of the foramen of Monro, and ΔEvans_total represents the net change in ventricular size over the observation period. A positive value indicates progressive ventricular dilation, whereas a negative value indicates ventricular regression. This metric was chosen over static hemorrhage-based scores (e.g., modified Graeb score) because it captures the dynamic trajectory of ventricular morphology, which is more directly reflective of evolving CSF dynamics and the pathophysiological process leading to chronic hydrocephalus. (2) Hematoma clearance rate: exponential decay fitting of basal cistern hematoma CT attenuation values to estimate half-life (15, 24). (3) Inflammatory burden trajectory: area under the curve (AUC) and moving-average slope for CRP and CSF protein (20). (4) ICP burden: cumulative number of days with ICP > 200 mmH_2_O and the coefficient of variation (CV) of ICP. (5) Other dynamic clinical features, including peak WBC count, mean neutrophil count, and the rate of CSF erythrocyte decline.

Because complete computation of these dynamic derived features depends on data from the first three weeks of hospitalization, a landmark prediction design was adopted, setting the prediction time point at day 21 after admission (i.e., the end of week 3). The intended clinical application scenario is as follows: after completion of the three-week assessment, all observational information accumulated to that time point is entered into the model, which outputs the predicted probability of SDHC occurring during the subsequent follow-up period (after day 21 through 180 days after onset), thereby supporting subsequent management decisions. For patients discharged before 3 weeks with partial missing dynamic time points, imputation based on within-training-set fitted methods was applied to address missing values.

### 2.5 Data preprocessing

#### 2.5.1 Missing value handling

A random seed of 42 was fixed throughout all analyses to ensure reproducibility. A stratified missing-value handling strategy was applied: (1) static baseline variables with a missing rate <10% were imputed using median values for continuous variables and mode values for categorical variables (25); variables with a missing rate ≥10% were excluded from subsequent modeling; (2) weekly aggregated dynamic variables were imputed using K-nearest neighbor imputation (KNNImputer, n_neighbors = 5) (26), which operates on inter-sample feature similarity rather than a strict temporal model. To prevent information leakage, all imputers were fitted exclusively within each training fold and subsequently applied to the corresponding validation fold and external validation data. A potential source of bias should be acknowledged: patients with milder clinical status may be more likely to be discharged before completion of the three-week observation window, so that dynamic-variable missingness is plausibly related to underlying disease severity rather than occurring completely at random (i.e., missing-not-at-random). Because KNN imputation relies on similarity among patients with comparable observed feature profiles, it may only partially mitigate this bias; if patients discharged early are under-represented among training cases with complete week-3 data, the estimated association between late-window dynamic features and SDHC risk could be attenuated. To assess the potential impact of this bias, a sensitivity analysis restricted to patients with complete three-week dynamic data is reported in Supplementary Table S1.

#### 2.5.2 Feature encoding and standardization

Nominal categorical variables (e.g., aneurysm location, treatment modality) were encoded using one-hot encoding; ordinal variables (e.g., Hunt-Hess grade, modified Fisher grade) were numerically encoded according to their clinical ranks. Within each training fold of cross-validation, continuous variables were standardized using Z-score normalization (mean = 0, standard deviation = 1); the corresponding validation fold was transformed using parameters learned from the training fold. For external validation, standardization parameters derived from the entire development cohort were applied to the external validation cohort to ensure consistency of the feature space.

### 2.6 Feature selection

To reduce dimensionality, eliminate redundancy, and improve model generalizability, an ensemble feature selection strategy was employed to screen core features from the candidate feature pool (static baseline features, clinical intervention variables, and derived dynamic features) (27). Feature selection was executed as part of the inner-loop procedure within nested cross-validation to prevent information leakage. The specific steps were as follows: (1) within each inner training fold, feature importance was assessed using Lasso regression (LassoCV), random forest classifier (RandomForestClassifier), and XGBoost classifier (XGBClassifier) separately; (2) the importance scores from each method were independently normalized; (3) for each feature, the mean of normalized scores across the three methods was computed as its ensemble importance score; and (4) features were ranked in descending order by ensemble importance score, and the top 10 features were selected to participate in model training for that fold. The final feature set was defined as the stable features selected with a frequency ≥80% across the outer cross-validation folds; the optimal model was subsequently retrained on the complete development cohort using the same procedure and applied to external validation. The ≥80% retention threshold was adopted as a conservative stability criterion, consistent with the general principle that features selected reproducibly across resampled folds are less likely to reflect fold-specific noise (27). The detailed results of the ensemble feature selection procedure, including the individual importance scores from each method, ensemble scores, and cross-fold selection frequencies, are presented in Supplementary Table S2 (individual and ensemble feature-importance scores and cross-fold selection frequencies for all candidate variables). The LASSO regularization path is shown in Supplementary Figure S1 (coefficient trajectories across the regularization parameter).

### 2.7 Model development and training

Three representative machine learning algorithms spanning major algorithmic paradigms were selected for comparison: logistic regression (generalized linear model, serving as the classical statistical benchmark), random forest (bagging-based ensemble method with inherent capability for modeling nonlinear interactions and handling class imbalance), and XGBoost (boosting-based ensemble method with demonstrated superior performance on structured tabular data). This selection covers the principal categories of supervised learning algorithms applicable to clinical prediction tasks with moderate sample sizes. Deep learning methods (e.g., neural networks) were not considered due to the limited sample size (*n* = 228), which is insufficient for reliable training of high-parameter models. Support vector machines were excluded due to their limited interpretability and lack of consistent superiority over ensemble tree methods in tabular data settings.

Three representative machine learning models were compared: (1) Logistic Regression—a classical statistical benchmark model with L2 regularization and balanced class weights (28). (2) Random Forest—a powerful ensemble tree model that handles class imbalance via class_weight = ‘balanced’ (29). (3) XGBoost—an efficient gradient-boosted tree model that addresses class imbalance through the scale_pos_weight parameter (30).

All models were trained and internally validated within a nested cross-validation (nested CV) framework (31). The outer loop used 5-fold cross-validation for unbiased performance estimation; the inner loop used 5-fold cross-validation for feature selection and hyperparameter tuning (e.g., tree depth, learning rate, minimum samples to split). The 5-fold configuration for both loops follows widely adopted practice in clinical prediction modeling with moderate sample sizes, balancing the bias–variance trade-off of performance estimation: fewer folds (e.g., 3-fold) yield less stable estimates because of smaller training partitions, whereas a larger number of folds (e.g., 10-fold) increases the computational burden of the nested procedure without a clear accuracy benefit at this sample size (31). This strategy effectively reduces the risk of overfitting and minimizes performance overestimation.

### 2.8 Model evaluation

#### 2.8.1 Internal validation

Given that SDHC is a relatively rare outcome, model performance was comprehensively evaluated across multiple dimensions—discrimination, classification performance, calibration, and clinical utility: (1) Discrimination: AUC-ROC and AUPRC; (2) Classification performance: at the prespecified decision threshold of 0.30 (selected because the consequences of missed SDHC diagnosis outweigh those of false-positive classification), accuracy, sensitivity, specificity, positive predictive value, negative predictive value, and F1 score were reported; (3) Calibration: calibration curves and Brier score were used to assess agreement between predicted probabilities and observed risks; Hosmer-Lemeshow test was used as supplementary analysis; (4) Clinical net benefit: decision curve analysis (DCA) was used to evaluate net benefit across risk thresholds; (5) Statistical comparison: ROC curves were constructed from patient-level predicted probabilities pooled from the outer 5-fold cross-validation, and AUC differences between models were compared using the DeLong method.

#### 2.8.2 External validation

The optimal model trained on the complete development cohort was directly applied to the external validation cohort from the Affiliated Hospital of Putian University. The same evaluation metrics as those used for internal validation were applied. Because internal and external validation cohorts originated from different patient populations, independent-sample AUC comparison methods were used to assess cross-center stability. Patients were further stratified into low- (<0.30), intermediate- (0.30–0.60), and high-risk (>0.60) groups based on predicted probabilities, and observed SDHC rates across groups were compared for gradation. Given the limited number of positive samples in the external validation cohort (*n* = 24), 95% confidence intervals were reported for all major performance metrics.

### 2.9 Model interpretability analysis

To enhance model transparency and clinical interpretability, SHAP (SHapley Additive exPlanations) analysis was applied to the best-performing model (32, 33). SHAP values were computed for each feature to quantify its marginal contribution to individual predictions. A global feature importance ranking (based on mean absolute SHAP values) and a SHAP beeswarm plot were presented to visually depict the direction and magnitude of feature effects.

### 2.10 Collinearity handling

Given the known clinical correlation between GCS score and Hunt-Hess grade (Spearman r = −0.68, *p* < 0.001), three variable inclusion strategies were evaluated in preliminary experiments: GCS alone, Hunt-Hess grade alone, and both variables combined. The combined inclusion yielded the best discriminative performance (AUC 0.894) compared with GCS alone (AUC 0.881) or Hunt-Hess grade alone (AUC 0.876). This finding suggests that, despite their correlation, the two variables capture distinct dimensions of neurological status: GCS primarily quantifies level of consciousness, whereas Hunt-Hess grade emphasizes clinical symptom severity. Both variables were therefore retained in the final model.

### 2.11 Statistical analysis and software implementation

All statistical analyses and machine learning modeling were performed in Python 3.9, primarily using pandas (1.5.3), numpy (1.24.3), scikit-learn (1.3.0), xgboost (1.7.6), shap (0.42.1), matplotlib (3.7.2), and seaborn (0.12.2). Continuous variables were compared using the independent-samples t-test or Mann–Whitney U test according to the Shapiro–Wilk normality test; categorical variables were compared using the chi-square test or Fisher’s exact test. AUC comparisons between models were performed using the DeLong method or the corresponding independent-sample comparison approach. All statistical tests were two-tailed, and a *p* value <0.05 was considered statistically significant.

## 3 Results

### 3.1 General characteristics

#### 3.1.1 Development cohort

After applying the inclusion and exclusion criteria, a total of 228 patients were enrolled in the development cohort. The median patient age was 60 years (IQR 52–68 years), and 120 patients (52.6%) were female. SDHC occurred in 50 patients (21.9%) during follow-up, whereas 178 patients (78.1%) did not develop SDHC. The median time from aSAH onset to VPS implantation in the SDHC group was 84 days (IQR 60–102 days). The median length of hospital stay was 24 days (IQR 19–32 days); 202 patients (88.6%) remained hospitalized at day 21, confirming the feasibility of the 21-day landmark prediction within this clinical setting. During the screening process, 5 patients (2.1%) who underwent VPS implantation within 21 days of admission were excluded per the predefined exclusion criteria. Similarly, 2 patients (1.9%) in the external validation cohort were excluded for the same reason.

Compared with the non-SDHC group (Supplementary Table S3, comparing baseline and dynamic characteristics between the SDHC and non-SDHC groups within the development cohort), patients in the SDHC group were significantly older (68.70 ± 11.16 vs. 60.08 ± 12.41 years), had higher Hunt-Hess grades (3.98 ± 1.04 vs. 2.94 ± 1.18), lower GCS scores (6.60 ± 2.86 vs. 10.72 ± 3.84), and higher modified Fisher scores (2.98 ± 1.10 vs. 2.56 ± 0.96); all differences were statistically significant (all *p* < 0.05). In addition, the SDHC group had significantly greater maximum basal cistern SAH thickness (27.04 ± 6.72 vs. 15.67 ± 7.02 mm), higher cumulative CSF drainage volume during the first 3 postoperative days (95.44 ± 40.01 vs. 68.40 ± 30.09 mL) and first 14 postoperative days (121.74 ± 45.21 vs. 90.81 ± 39.10 mL), higher mean ICP during week 2 (188.00 ± 57.32 vs. 163.05 ± 49.02 mmH₂O) and week 3 (169.48 ± 57.64 vs. 134.42 ± 51.04 mmH₂O) after surgery, higher Evans index at week 1 (0.29 ± 0.05 vs. 0.25 ± 0.04) and week 2 (0.27 ± 0.03 vs. 0.26 ± 0.02), and higher mean WBC counts during weeks 1, 2, and 3 after surgery (all *p* < 0.05).

With respect to categorical variables, the SDHC group had significantly higher proportions of patients with smoking history (30.0% vs. 6.8%), acute hydrocephalus (20.0% vs. 1.7%), intraventricular hemorrhage (62.0% vs. 39.5%), decompressive craniectomy (24.0% vs. 2.8%), re-bleeding (14.0% vs. 2.8%), seizure (14.0% vs. 2.8%), periventricular edema (38.0% vs. 9.0%), and antifibrinolytic drug use (28.0% vs. 10.2%) compared with the non-SDHC group (all *p* < 0.05). Other variables—including sex, comorbidities (hypertension, diabetes mellitus, atrial fibrillation), alcohol use history, aneurysm laterality, aneurysm location, maximum diameter, ICU length of stay, Evans index at week 3, intracranial infection, statin use, weekly changes in basal cistern CT attenuation, neutrophil ratio, CRP, PCT, CSF protein, CSF erythrocyte count, and CSF leukocyte count—did not differ significantly between the two groups (all *p* > 0.05).

#### 3.1.2 External validation cohort

The external validation cohort comprised 102 patients with aSAH, with a median age of 58 years (IQR 50–67 years) and 57 female patients (55.9%). The SDHC incidence was 23.5% (24/102), which did not differ significantly from the development cohort rate of 21.9% (χ^2^ = 0.11, *p* = 0.740). Comparison of baseline characteristics between the two cohorts (Table 1) revealed no statistically significant differences in age, sex, history of hypertension, history of diabetes mellitus, admission GCS score, Hunt-Hess grade, modified Fisher grade, aneurysm location distribution, or treatment modality (all *p* > 0.05), indicating good comparability between the two cohorts.

**Table 1:** Comparison of baseline characteristics between the development and external validation cohorts.

Variable	Development cohort (*n* = 228)	External validation cohort (*n* = 102)	*p* value
Age, years, median (IQR)	60 (52–68)	58 (50–67)	0.312ᵃ
Female, *n* (%)	120 (52.6)	57 (55.9)	0.578ᵇ
History of hypertension, *n* (%)	118 (51.8)	49 (48.0)	0.534ᵇ
History of diabetes mellitus, *n* (%)	38 (16.7)	15 (14.7)	0.656ᵇ
Admission GCS score, mean ± SD	9.82 ± 4.02	9.56 ± 3.89	0.578ᶜ
Hunt-Hess grade, mean ± SD	3.17 ± 1.21	3.24 ± 1.18	0.621ᵃ
Modified Fisher grade, mean ± SD	2.65 ± 1.00	2.71 ± 0.98	0.612ᵃ
Anterior circulation aneurysm, *n* (%)	156 (68.4)	68 (66.7)	0.754ᵇ
Endovascular treatment, *n* (%)	142 (62.3)	67 (65.7)	0.552ᵇ
SDHC incidence, *n* (%)	50 (21.9)	24 (23.5)	0.740ᵇ
Evans_total (Ventricle Ratio Wk3 - Wk1)	0.02 ± 0.04	0.02 ± 0.05	0.512ᵃ
Coefficient of Variation of ICP (Wk 1–3)	0.18 ± 0.06	0.19 ± 0.07	0.235ᵃ
14-day cumulative CSF drainage volume (mL)	98.5 ± 41.2	101.2 ± 43.5	0.578ᵃ
3-day cumulative CSF drainage volume (mL)	74.2 ± 35.1	76.5 ± 33.8	0.556ᵃ
Inflammatory burden (based on CRP Wk 1–3)	45.2 ± 15.3	46.8 ± 16.1	0.367ᵃ
Hematoma clearance half-life (days)	4.2 ± 1.5	4.1 ± 1.6	0.560ᵃ
Peak WBC count within 3 weeks (×10^9^/L)	12.5 ± 4.2	12.8 ± 4.5	0.542ᵃ

ᵃMann–Whitney U test; 𝑼hi-square test; ᶜIndependent-samples *t*-test. IQR, interquartile range; SD, standard deviation; GCS, Glasgow Coma Scale; SDHC, shunt-dependent hydrocephalus.

### 3.2 Discriminative performance and statistical comparison of predictive models (internal validation)

**Figure 2:**
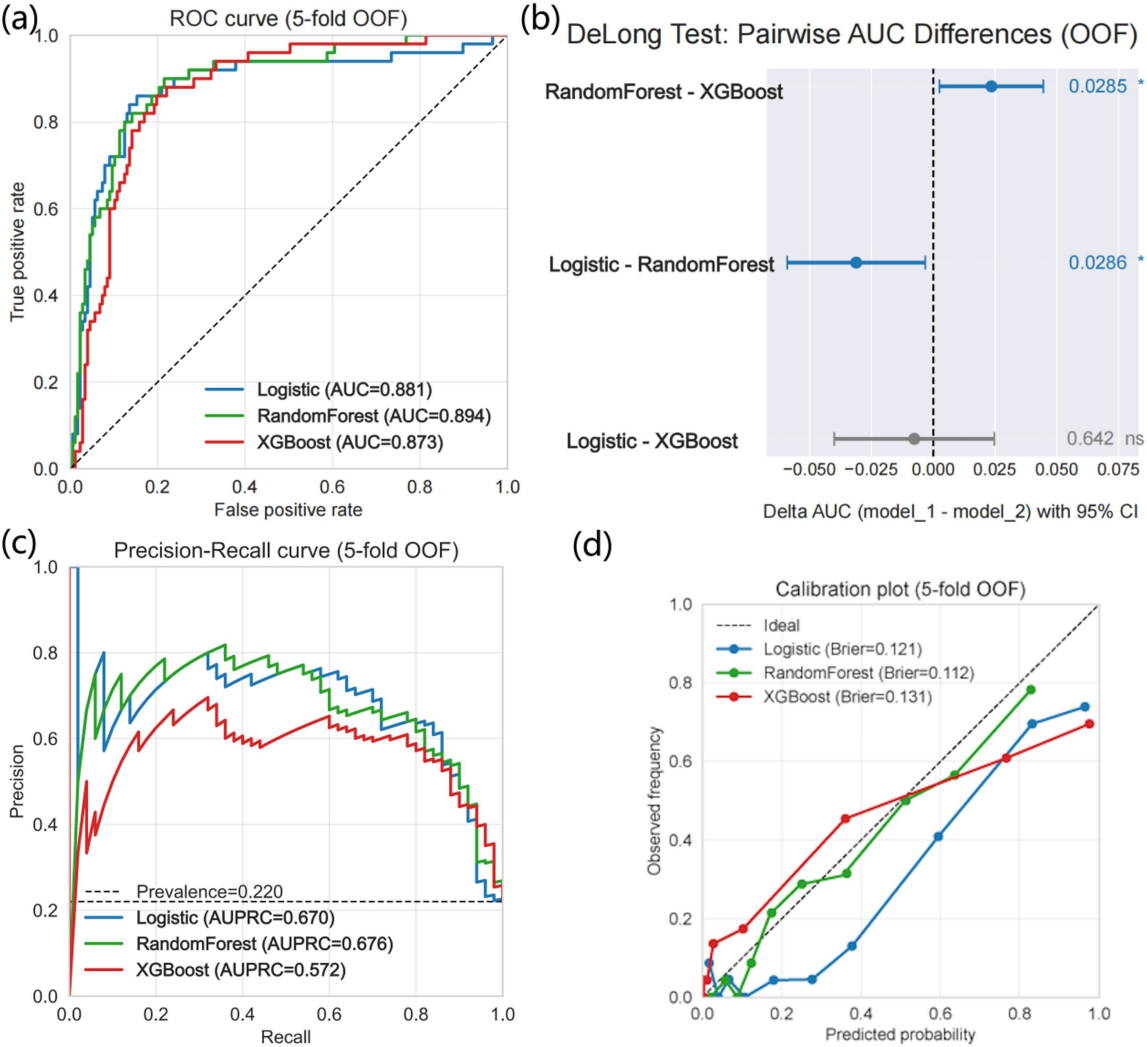
Comparison of discrimination and calibration performance among three prediction models (internal validation). **(a,b)** Receiver operating characteristic (ROC) curves based on pooled predicted probabilities from 5-fold cross-validation. The random forest (AUC = 0.894) significantly outperformed logistic regression (AUC = 0.881; ΔAUC = 0.013, DeLong *p* < 0.05) and XGBoost (AUC = 0.873; ΔAUC = 0.021, DeLong *p* < 0.05). **(c)** Precision-recall curves (baseline positive rate = 0.220). Logistic regression and random forest showed similar AUPRC values (0.678 vs. 0.676, *p* = 0.91), both significantly outperforming XGBoost (0.572). **(d)** Brier score comparison. Random forest achieved the lowest calibration error (0.112), followed by logistic regression (0.121) and XGBoost (0.131).

In the internal validation with 5-fold nested cross-validation, the random forest model demonstrated the best overall discriminative performance (Figures 2a,b). Its AUC of 0.894 (95% CI, 0.869–0.935) was superior to that of the logistic regression model (0.881; 95% CI, 0.834–0.902) and the XGBoost model (0.873; 95% CI, 0.848–0.916). DeLong method comparisons indicated that the AUC differences between random forest and both logistic regression and XGBoost were statistically significant (both *p* < 0.05), whereas the difference between logistic regression and XGBoost was not significant (*p* = 0.642).

In the setting of class imbalance, AUPRC further corroborated these findings: the AUPRC values of logistic regression and random forest were 0.678 and 0.676, respectively—comparable to each other and both superior to that of XGBoost (0.572; Figure 2c). Regarding calibration, the random forest achieved the lowest Brier score of 0.112 (95% CI, 0.098–0.126), outperforming logistic regression (0.121) and XGBoost (0.131; Figure 2d). Furthermore, the calibration curve of the random forest demonstrated good agreement between predicted probabilities and observed risks, with the Hosmer-Lemeshow test indicating no significant miscalibration (χ^2^ = 6.32, *p* = 0.612).

### 3.3 Ablation analysis: contribution of dynamic features

To quantify the incremental contribution of dynamic features, an ablation analysis was performed by constructing a random forest model using only the six static baseline features (GCS score, Hunt-Hess grade, age, periventricular edema, decompressive craniectomy, and acute hydrocephalus) under the same nested cross-validation framework. The static-only model achieved an internal validation AUC of 0.831 (95% CI, 0.790–0.872) and an external validation AUC of 0.798 (95% CI, 0.722–0.874). Compared with the full model incorporating dynamic features, the addition of dynamic variables yielded a statistically significant improvement in internal validation AUC (ΔAUC = +0.063, DeLong *p* = 0.028) and external validation AUC (ΔAUC = +0.069). The AUPRC improved from 0.548 to 0.676, and the Brier score decreased from 0.141 to 0.112, indicating enhanced discrimination and calibration. Furthermore, the cumulative SHAP contribution of the four dynamic features (ΔEvans_total, ICP coefficient of variation, 14-day CSF drainage volume, and peak WBC count) accounted for 22.2% of the total mean absolute SHAP values across all features. These findings confirm that, while static baseline variables remain the dominant predictors, dynamic features provide substantial and statistically significant incremental predictive value by capturing ongoing pathophysiological evolution not reflected in admission-time assessments.

### 3.4 Classification performance of models at the decision threshold (internal validation)

**Figure 3:**
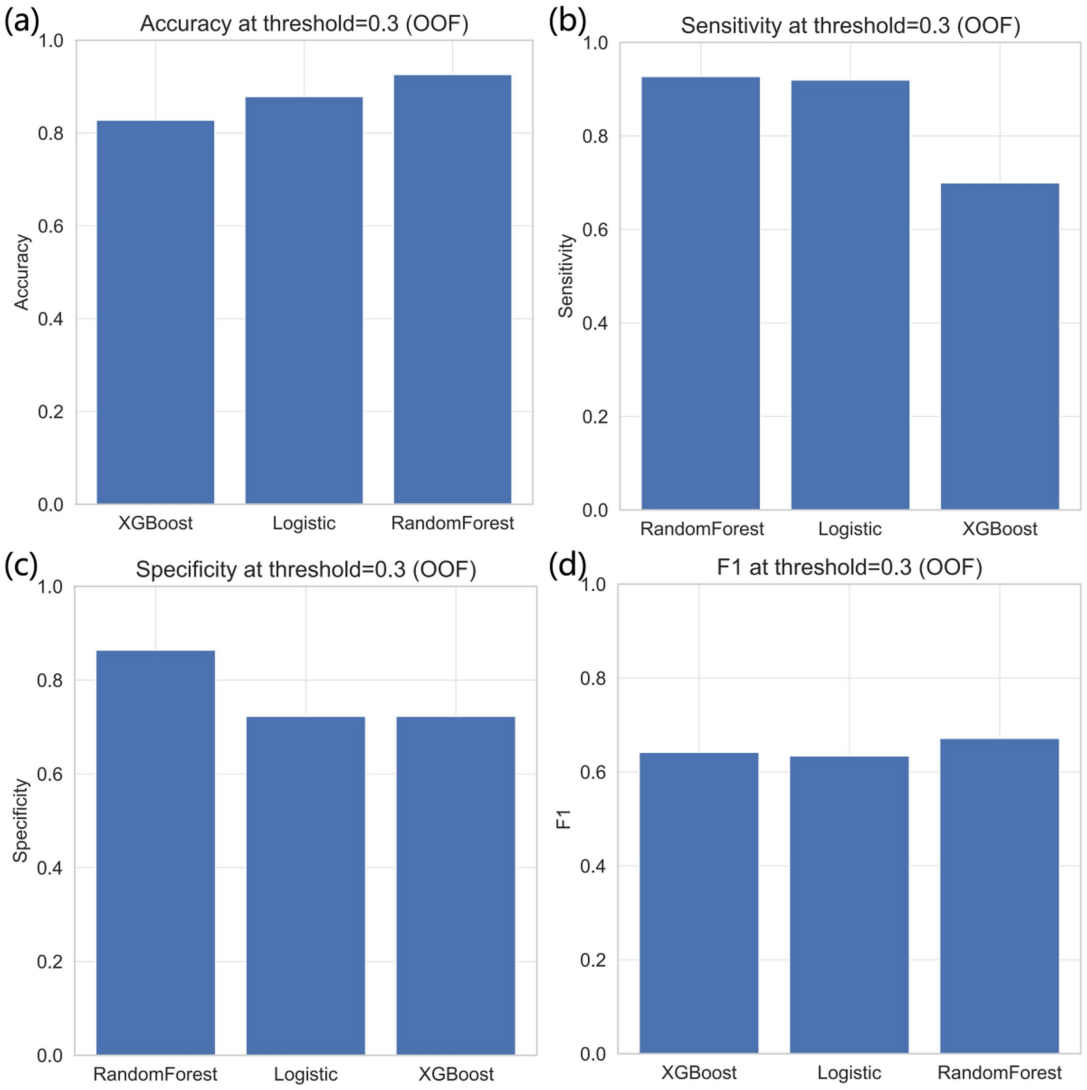
Classification performance metrics of each model at a threshold of 0.30 (internal validation). **(a)** Accuracy: Random forest achieved the highest accuracy (0.892). **(b)** Sensitivity: Both random forest and logistic regression exceeded 0.90, while XGBoost showed lower sensitivity (0.71). **(c)** Specificity: Random forest (0.84) was significantly higher than logistic regression and XGBoost. **(d)** F1 score: Random forest achieved the best F1 score (0.67), reflecting its superior balance between precision and recall.

At the prespecified decision threshold of 0.30, the classification performance metrics for each model are presented in Figure 3. The random forest model achieved the highest accuracy (0.892), outperforming logistic regression and XGBoost (Figure 3a). In terms of sensitivity, both random forest and logistic regression exceeded 0.90, indicating that both models could correctly identify the vast majority of SDHC patients; by contrast, XGBoost had a sensitivity of approximately 0.71, reflecting higher miss rates (Figure 3b). With respect to specificity, random forest also performed best (0.84), indicating a superior ability to reduce false-positive results (Figure 3c). Furthermore, the random forest achieved the highest F1 score (0.67), reflecting an optimal balance between precision and recall (Figure 3d).

### 3.5 Operational performance and net benefit at the clinical decision threshold (internal validation)

**Figure 4:**
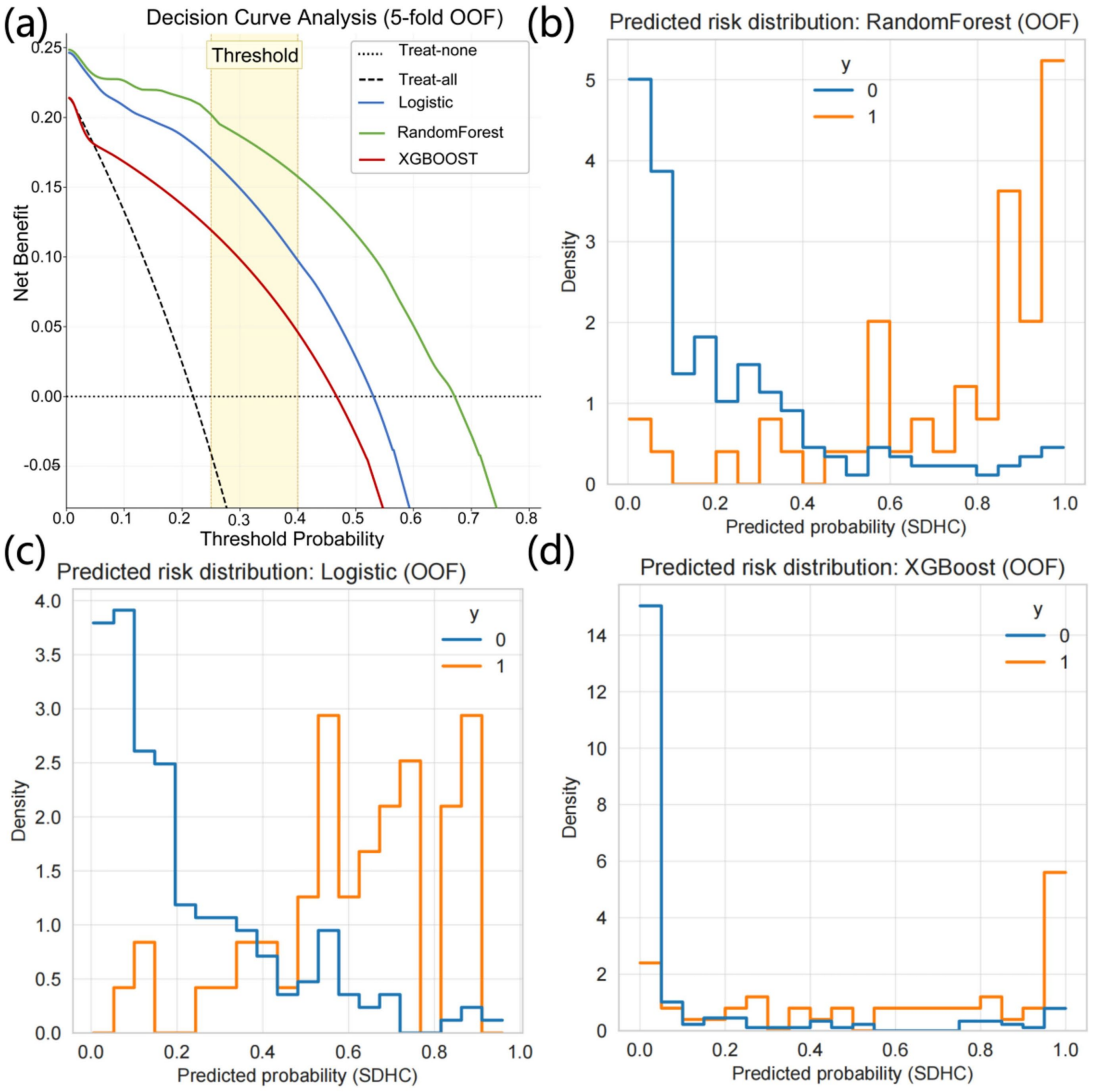
Clinical decision analysis and predicted risk distribution (internal validation). **(a)** Decision curve analysis (DCA). All models provided positive net benefit within the threshold probability range of 0.10–0.50; random forest demonstrated the highest net benefit in the clinically relevant range of 0.25–0.40. **(b–d)** Histograms of predicted risk distribution (5-fold cross-validation). All three models exhibited bimodal distributions; random forest showed the clearest separation between low-risk (0–0.3) and high-risk (0.6–1.0) groups, with the lowest proportion of intermediate-risk samples, indicating superior risk stratification capability.

DCA showed that all three models provided positive net benefit across a threshold probability range of 0.10 to 0.45 (Figure 4a). Within the clinically more common threshold range of 0.25 to 0.40, the net benefit of random forest consistently exceeded that of logistic regression and XGBoost. For example, at a threshold of 0.30, the net benefits of random forest, logistic regression, and XGBoost were 0.182, 0.150, and 0.100, respectively. As the threshold increased further, the net benefits of the models gradually decreased, and at higher thresholds the ‘treat-none’ strategy tended to become dominant. Figures 4b–d display the predicted risk distributions across the three models under 5-fold cross-validation. All three models exhibited a degree of bimodal distribution; however, random forest showed the most pronounced separation between the low-risk zone (0–0.30) and the high-risk zone (0.60–1.00), with the smallest proportion of samples in the intermediate-risk range, indicating superior risk stratification capability and greater suitability for clinical stratified management and decision-making.

### 3.6 External validation results

#### 3.6.1 Discriminative performance

**Figure 5:**
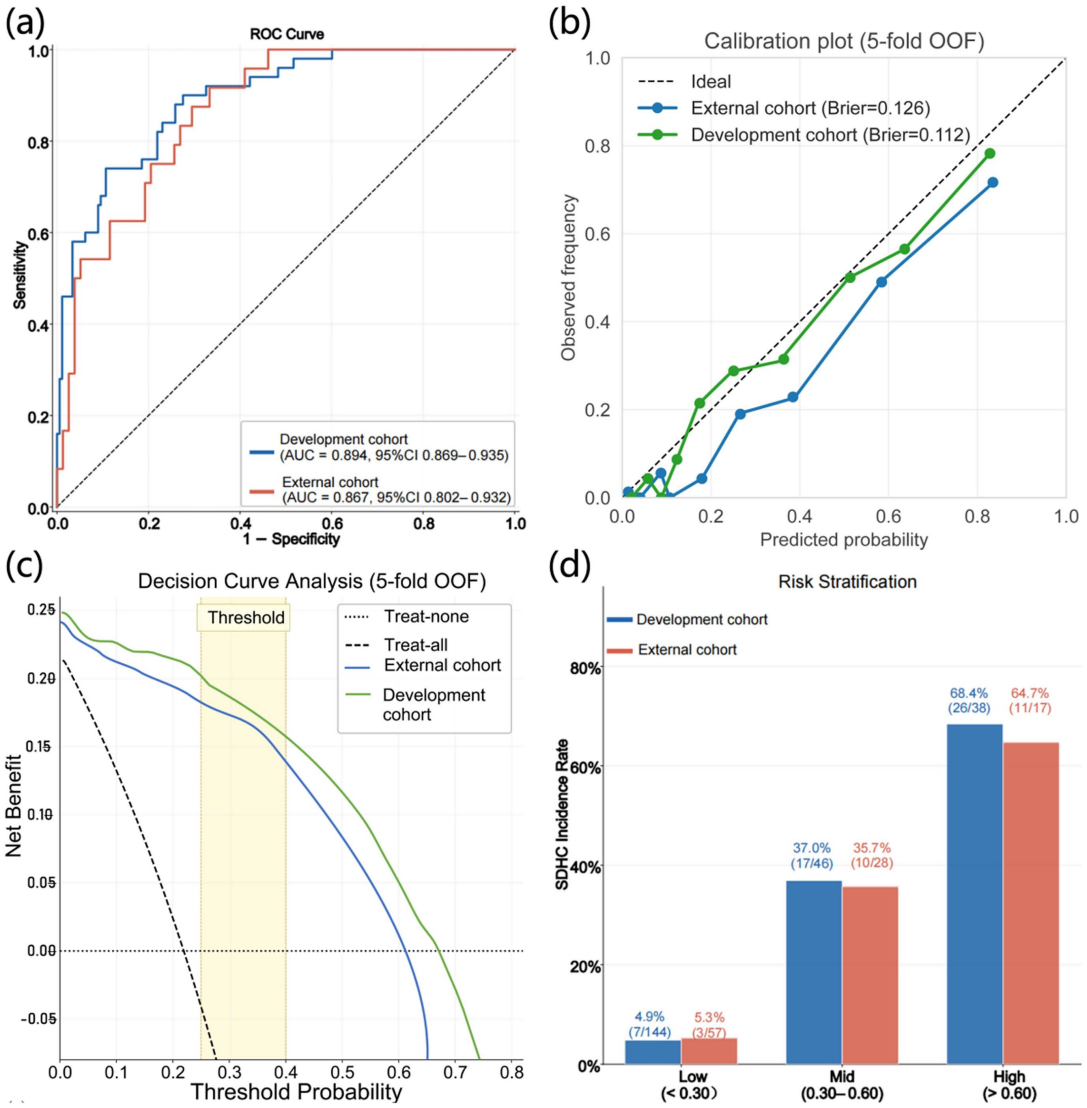
External validation results of the random forest model. **(a)** ROC curve comparison: ROC curves of the development cohort (AUC = 0.894) and external validation cohort (AUC = 0.867); the difference was not statistically significant (DeLong *p* = 0.263). **(b)** Calibration curve for the external validation cohort: Predicted probabilities showed good agreement with observed frequencies. **(c)** Decision curve analysis for the external validation cohort: The model provided positive net benefit within the threshold range of 0–0.6. **(d)** Risk stratification validation: Comparison of observed SDHC rates increased stepwise across low- (5.3%), intermediate- (35.7%), and high-risk (64.7%) strata in the external validation cohort, closely matching the development cohort pattern (4.9, 37.0, 68.4%; trend *p* < 0.001 in both cohorts).

When the final random forest model trained on the development cohort was applied to the external validation cohort, the model retained good generalizability (Figure 5a). The AUC in the external validation cohort was 0.867 (95% CI, 0.802–0.932), representing only a marginal decline from the internal validation AUC of 0.894, and the difference was not statistically significant (*p* = 0.263), indicating satisfactory cross-center stability. The AUPRC for the external validation cohort was 0.641; in conjunction with a positive rate of 23.5% in this cohort, this result suggests that the model maintained reasonably robust identification of positive events.

#### 3.6.2 Classification performance metrics

At the prespecified threshold of 0.30, the classification performance metrics in the external validation cohort are presented in Table 2. The accuracy was 0.863, sensitivity 0.875 (21/24 SDHC patients correctly identified), specificity 0.859 (67/78 non-SDHC patients correctly classified), and F1 score 0.636. The declines in all metrics relative to internal validation remained within acceptable ranges (AUC decrease <0.03; other metrics decrease <0.05), consistent with the expected performance attrition during external validation of predictive models.

**Table 2:** Performance comparison of the random forest model between the development and external validation cohorts.

Metric	Development cohort (internal validation)	External validation cohort
True positives (TP)	46	21
False negatives (FN)	4	3
True negatives (TN)	150	67
False positives (FP)	28	11
Accuracy	0.892 (0.805–0.903)	0.863 (0.780–0.921)
Sensitivity	0.920 (0.812–0.972)	0.875 (0.677–0.965)
Specificity	0.843 (0.781–0.893)	0.859 (0.763–0.923)
Positive predictive value (PPV)	0.622 (0.500–0.731)	0.656 (0.471–0.804)
Negative predictive value (NPV)	0.974 (0.934–0.991)	0.957 (0.879–0.987)
F1 score	0.670	0.750

#### 3.6.3 Calibration assessment

The Brier score in the external validation cohort was 0.126 (95% CI, 0.098–0.154), comparable to that of the development cohort, indicating that model calibration was preserved in the independent population (Figure 5b). The calibration curve of the external validation cohort demonstrated generally good agreement between predicted probabilities and observed frequencies in the low- and intermediate-risk ranges, with no systematic overestimation or underestimation observed in the high-risk range (>0.60). The Hosmer-Lemeshow test yielded χ^2^ = 8.45, *p* = 0.391, indicating no significant calibration deviation.

#### 3.6.4 Decision curve analysis

DCA in the external validation cohort (Figure 5c) showed that the random forest model provided positive net benefit across a threshold probability range of 0 to 0.60. Within the clinically common threshold range of 0.25 to 0.40, the net benefit of the model was comparable to that of the development cohort (at threshold 0.30: external validation net benefit 0.165 vs. development cohort 0.182), confirming the clinical utility of the model in an independent population.

#### 3.6.5 Risk stratification stability

Patients in the external validation cohort were stratified into low-risk (<0.3), intermediate-risk (0.3–0.6), and high-risk (>0.6) groups based on predicted probabilities; the observed SDHC rates were 5.3% (3/57), 35.7% (10/28), and 64.7% (11/17), respectively, demonstrating a clear stepwise increasing trend (trend χ^2^ = 28.6, *p* < 0.001; Figure 5d; Table 3). This pattern was highly consistent with that of the development cohort, indicating stable risk stratification capability of the model.

**Table 3:** Risk stratification comparison between the development and external validation cohorts.

Risk stratum	Development cohort, *n* (%)	SDHC, *n*/*N* (%)	External validation cohort, *n* (%)	SDHC, *n*/*N* (%)
Low risk (<0.3)	144 (63.2%)	7/144 (4.9%)	57 (55.9%)	3/57 (5.3%)
Intermediate risk (0.3–0.6)	46 (20.2%)	17/46 (37.0%)	28 (27.5%)	10/28 (35.7%)
High risk (>0.6)	38 (16.7%)	26/38 (68.4%)	17 (16.7%)	11/17 (64.7%)
Total	228 (100%)	50/228 (21.9%)	102 (100%)	24/102 (23.5%)
Trend test	*p* < 0.001		*p* < 0.001	

### 3.7 Model interpretability and quantitative contributions

**Figure 6:**
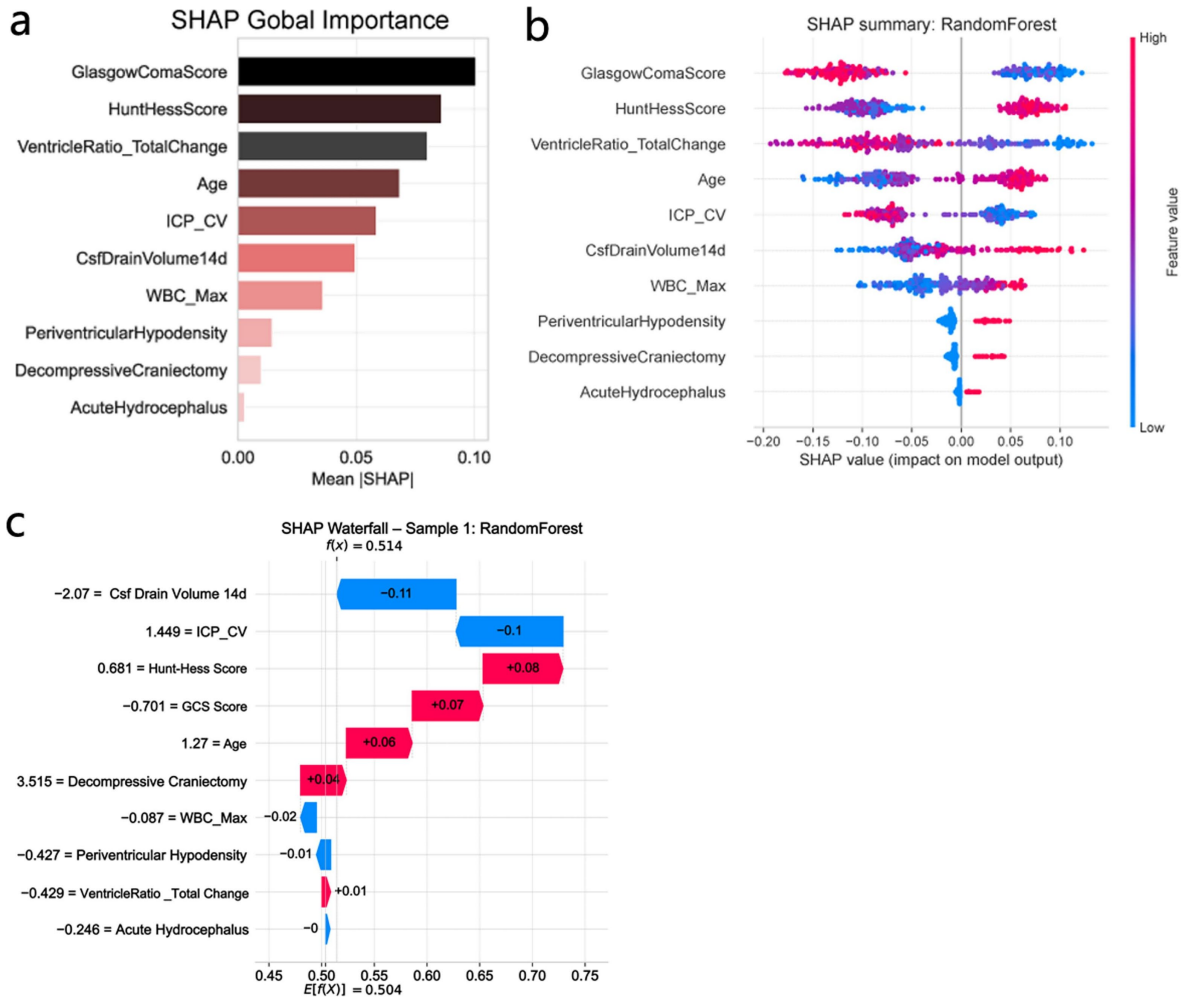
Feature importance and interpretability analysis of the random forest model. **(a)** Global feature importance ranking based on SHA*p* values (top 10 features). GCS score (0.100) and Hunt-Hess grade (0.080) were the most influential predictors, while coefficient of variation of ICP (0.058) and total change in Evans index (0.075) highlighted the important contribution of dynamic features. **(b)** SHAP summary (beeswarm) plot. Each point represents one patient; color indicates the feature value (red = high, blue = low) and horizontal position indicates the SHAP value (impact on predicted SDHC risk). Lower GCS score, higher Hunt-Hess grade, older age, a greater positive change in Evans index (progressive ventricular dilation), higher ICP coefficient of variation, higher postoperative 14-day CSF drainage volume, higher peak WBC count, and the presence of periventricular edema, decompressive craniectomy, or acute hydrocephalus were each associated with more positive SHAP values, indicating increased predicted SDHC risk. **(c)** SHAP waterfall plot for a representative case. The plot illustrates the cumulative contribution of each feature to the individual predicted risk; red indicates increased risk, blue indicates decreased risk.

In the best-performing random forest model, the global feature importance ranking based on SHAP values was as follows (Figure 6a): admission GCS score (0.100), Hunt-Hess grade (0.080), total change in ventricular fraction (0.075), age (0.062), ICP coefficient of variation (0.058), postoperative 14-day CSF drainage volume (0.049), peak peripheral WBC count over 3 weeks (0.040), periventricular edema (0.022), decompressive craniectomy (0.018), and acute hydrocephalus (0.009). The SHAP beeswarm plot (Figure 6b) revealed the directional associations between each feature and SDHC risk: lower GCS score corresponded to more positive SHAP values, indicating higher risk; greater ICP coefficient of variation was associated with more positive SHAP values, indicating higher risk. Higher Hunt-Hess grade, older age, higher postoperative 14-day drainage volume, higher peak WBC count, and the presence of periventricular edema, decompressive craniectomy, or acute hydrocephalus were all associated with higher predicted SDHC risk. A positive total change in Evans index (i.e., progressive ventricular dilation) was associated with increased SDHC risk. Representative waterfall plots (Figure 6c) intuitively display the direction and magnitude of each feature’s contribution to individual predicted risks. As a supplementary analysis, a multivariable logistic regression was performed to provide adjusted odds ratios for the 10 selected features (Supplementary Table S4, multivariable logistic regression odds ratios for the 10 selected features). The results were broadly consistent with the SHAP-based importance ranking, with GCS score, Hunt-Hess grade, and decompressive craniectomy demonstrating the strongest independent associations with SDHC. There was no statistically significant difference in model predictive performance between the two surgical subgroups, confirming that the model remains stable across different treatment strategies (Supplementary Table S5). The trends of SHAP contributions for core pathophysiological features (such as ventricular dilation and ICP fluctuations) in males and females are highly consistent. This confirms that gender-related risk information has been “masked” by these more dynamic pathophysiological indicators (Supplementary Table S6).

## 4 Discussion

Based on a dual-center retrospective cohort, this study integrated static baseline data, dynamic in-hospital monitoring data, and clinical intervention information from patients with aSAH to develop and externally validate a machine learning model for predicting SDHC risk. The results showed that the random forest model achieved the best overall performance in internal validation (AUC 0.894) and maintained satisfactory discrimination and calibration in an independent external validation cohort (AUC 0.867). Moreover, the model demonstrated stable net benefit within clinically relevant threshold ranges and was capable of stratifying patients into low-, intermediate-, and high-risk tiers, suggesting its potential clinical utility.

### 4.1 Comparison with prior studies

Comparison with prior literature can be organized along three dimensions. First, relative to traditional static scoring systems such as SDASH and CHESS (AUC approximately 0.77–0.82) (6, 13), the present model achieved higher discrimination (AUC 0.894 internally, 0.867 externally). Second, relative to single-time-point or static-feature machine learning models, a recent systematic review and meta-analysis pooling six ML-based SDHC prediction studies (2096 patients) reported a pooled AUC of 0.83 (95% CI, 0.81–0.84) (34), and an earlier single-center ML study restricted to admission and early laboratory variables reported an AUC in the 0.80–0.81 range (6); the present dynamic-feature model exceeded these benchmarks in both internal and external validation. Third, relative to foreign studies that have explicitly incorporated continuous or dynamic ICU monitoring data, a recurrent neural network model trained on routine ICU time-series data from 602 aSAH patients demonstrated the feasibility of dynamic-data-driven shunt-dependency prediction (35), and a 2024 multivariable model based on a 510-patient cohort similarly highlighted the value of longitudinally collected clinical variables (36). Compared with these dynamic-data approaches, the present study additionally contributes an explicit ablation quantification of the incremental value of dynamic features (ΔAUC = 0.063) and independent cross-center external validation, both of which were not concurrently reported in the studies above. Notably, the degree of performance attenuation during external validation (ΔAUC = 0.027) was substantially smaller than the typical range reported in the literature (0.05–0.10) (13), indicating robust model stability, possibly related to the effective prevention of overfitting at the internal validation stage achieved through the rigorous nested cross-validation strategy. Notably, an ablation analysis demonstrated that the full model integrating dynamic features significantly outperformed a static-only model (AUC 0.894 vs. 0.831, *p* = 0.028), confirming that the dynamic variables—rather than the machine learning algorithm alone—were a key driver of the performance improvement.

### 4.2 Clinical significance of core predictors

Neurological status at admission (low GCS score, high Hunt-Hess grade) is well established as a strong predictor of SDHC (8, 37), and the present study confirmed that these two features were the most important contributors. Age was also identified as a key predictor, possibly reflecting impaired arachnoid granulation absorption function, reduced brain compliance, and diminished recovery capacity in elderly patients. Intraventricular hemorrhage (IVH) is a well-established risk factor for SDHC and a core component of several predictive scores (e.g., Graeb score, MAI score) (11, 12). In the present study, the total change in Evans index over three weeks (ΔEvans_total) emerged as the third most important feature. This variable directly quantifies the degree of progressive ventricular dilation during the acute-to-subacute phase—a morphological consequence that may result from multiple upstream mechanisms including IVH-related CSF pathway obstruction, arachnoid granulation fibrosis, and impaired CSF absorption. Notably, ΔEvans_total captures a distinct dimension of information (dynamic ventricular trajectory) compared with IVH itself (a static event), and the two variables are complementary rather than redundant. Mechanistically, these three dynamic features can be conceptualized as sequential markers along a common pathophysiological cascade. Early intraventricular and subarachnoid blood breakdown provokes an inflammatory response in the subarachnoid space and choroidal/ependymal lining, reflected clinically by the cumulative WBC and inflammatory trajectory; this inflammatory milieu is thought to promote fibrosis and scarring of the arachnoid granulations, impairing CSF reabsorption. Impaired reabsorption manifests physiologically as unstable, poorly buffered intracranial pressure-captured by the ICP coefficient of variation-and, morphologically, as progressive enlargement of the ventricular system, captured by ΔEvans_total. Cumulative CSF drainage volume can be viewed as a clinical proxy for the degree to which a patient’s intracranial compliance already depends on external diversion, i.e., an early behavioral signal of eventual shunt dependency. Under this framework, ΔEvans_total and ICP variability are not merely statistically associated with SDHC but represent measurable downstream consequences of the same fibrosis-mediated impairment of CSF dynamics that ultimately necessitates permanent shunting.

More importantly, several dynamic and intervention-related variables were included in the final model. The total change in Evans index reflects the morphological evolution of the ventricular system during the first three weeks of hospitalization, and its importance supports the concept that ‘sustained ventricular dilation’ is a more informative indicator of SDHC risk than measurement at a single time point (23). The inclusion of ICP coefficient of variation—rather than mean ICP alone—among the top features suggests that instability in ICP regulation may more sensitively reflect abnormal CSF dynamics than the absolute ICP level. Postoperative 14-day CSF drainage volume, as an important predictor (6), may reflect the degree of physiological dependence on CSF diversion. Decompressive craniectomy (DC) has been confirmed as a significant risk factor for SDHC (OR >3) in multiple studies (2, 14), possibly related to changes in CSF dynamics and brain tissue collapse following DC (2). Furthermore, the positive association between peak peripheral WBC count over 3 weeks and SDHC risk is consistent with prior findings implicating infection/inflammation as risk factors (2, 8), suggesting that systemic inflammation may contribute to the pathogenesis of chronic hydrocephalus by promoting fibrosis of the arachnoid granulations.

Notably, one systematic review reported that female sex may be associated with higher SDHC risk (38), yet sex was not among the top 10 most important features in the final feature set of the present study. Several plausible explanations may account for this finding. First, the sample size of this study (*n* = 228) may have been insufficient to achieve adequate statistical power for detecting sex as a predictor of moderate effect size. Second, as this study was conducted exclusively in Chinese patients, sex-related SDHC mechanisms or baseline characteristic distributions may differ from those in Western populations, and the generalizability of the findings warrants validation in larger studies. Third, within the multivariate competitive framework of random forest, the risk information associated with sex may have been captured by pathophysiological indicators with stronger direct predictive power—such as ICP dynamics and ventricular dilation—resulting in a relatively marginal incremental contribution of sex. These explanations remain inferential; a formal subgroup analysis stratifying SHAP contributions by sex, reported in Supplementary Table S6, is required to determine whether sex-associated risk is genuinely absorbed by correlated pathophysiological indicators or whether it reflects limited statistical power in this cohort.

### 4.3 Clinical translation value

The model developed in this study has clear potential for clinical translation. The prediction window is set at day 21 after admission. We acknowledge that clinical practices regarding the timing of shunt dependency evaluation vary across centers. In some centers, particularly those adopting the EVD weaning trial protocol, shunt dependency may be assessed and VPS implantation may occur before day 21 (39, 40). However, in the two participating Chinese neurosurgical centers, the standard practice following aSAH involves extended observation with conversion from EVD to lumbar drainage rather than early EVD weaning trials, resulting in median hospitalization durations of 18–28 days. Consequently, the 21-day landmark is well aligned with the clinical workflow of these centers. Moreover, this time point is methodologically essential for the computation of derived dynamic features (e.g., three-week Evans index trajectory, ICP coefficient of variation, and inflammatory burden AUC) that form the basis of the model’s incremental predictive value. In our development cohort, only 5 patients (2.1%) underwent VPS before day 21, and these cases were predominantly attributable to acute refractory hydrocephalus with distinct pathophysiological mechanisms. For centers employing early EVD weaning protocols, the present model may serve as a complementary risk stratification tool that integrates cumulative dynamic information beyond the scope of the acute-phase EVD weaning assessment. Future studies should explore models with earlier or flexible prediction windows to accommodate diverse clinical workflows.

Early and reliable prediction of SDHC enables clinicians to proactively identify high-risk patients, plan treatment strategies earlier, intensify monitoring, and potentially reduce hospitalization duration and the risk of complications through optimized management (6). The high discriminative performance (AUC 0.867–0.894) and clear three-tier risk stratification (SDHC rates of approximately 5, 37, and 67% in the low-, intermediate-, and high-risk groups, respectively) allow more precise identification of individuals requiring close attention than conventional scoring systems. The DCA demonstrated unequivocal net benefit within the clinically reasonable threshold range of 0.25 to 0.40, indicating that use of this model to guide follow-up and decision-making in clinical practice would confer genuine patient benefit. Early identification of high-risk patients can facilitate timely implementation of individualized monitoring protocols, rational planning of CSF drainage strategies, and potentially shorter hospitalization through optimized perioperative management (6). Future efforts should focus on prospective, multicenter external validation of this model in diverse settings to confirm its generalizability (13). Additionally, integrating the model’s predicted outputs with specific clinical phenotypes of individual patients—such as those primarily presenting with chronic intracranial hypertension or delayed neurological recovery—may further enable individualized patient management and surgical decision-making (2), with the ultimate goal of improving long-term functional outcomes in patients with aSAH.

Regarding the concern of cross-center standardization of dynamic variables, it is noteworthy that the dynamic variables used in this model—including Evans index, ICP, WBC count, and CSF drainage volume—are routine clinical measurements available in virtually all neurosurgical centers. The derived features were intentionally designed using relative or normalized metrics (e.g., coefficient of variation rather than absolute ICP values) to enhance cross-center consistency. The narrow AUC gap between internal and external validation (ΔAUC = 0.027) provides empirical evidence that these dynamic features generalize well across centers with different clinical practices.

Given substantial disparities in equipment and staffing across Chinese hospital tiers, clinical translation should be tiered accordingly. In primary and secondary hospitals, where continuous ICP monitoring or dedicated neuro-critical-care infrastructure may be limited, the model can be implemented using its minimum viable feature subset-admission GCS score, Hunt-Hess grade, age, weekly Evans index (obtainable from routine follow-up CT already performed per local protocol), and cumulative CSF drainage volume - to provide a low-cost triage tool that flags high-risk patients for early transfer or telemedicine consultation. In tertiary neurosurgical centers with full multimodal monitoring capability, the complete feature set, including ICP coefficient of variation and inflammatory-burden trajectories, can be deployed to support fine-grained risk stratification, individualized CSF-drainage strategies, and shared decision-making regarding the timing of shunt evaluation.

### 4.4 Strengths of the study

The strengths of this study include the following. First, a rigorous nested cross-validation strategy was employed, with feature selection and hyperparameter tuning performed entirely within the inner loop to effectively prevent information leakage and overfitting; this rigorous internal validation is critical for assessing the reliability of predictive models (13). Second, cross-center generalizability was demonstrated through an independent external validation cohort (Affiliated Hospital of Putian University, *n* = 102), which is a key step for clinical translation of predictive models and represents an important advantage of this study relative to most prior studies that relied solely on internal validation (6, 13). Third, this study transcended the static prediction framework by using derived feature engineering to transform temporal dynamics of ICP, imaging, and laboratory findings into quantifiable indicators with clear clinical meaning; this may be a key driver of the improvement in model performance, and is consistent with the approach of Frey et al. (6) Fourth, the comprehensive use of calibration curves, DCA, and the SHAP interpretability framework enabled evaluation not only of discriminative ability but also of calibration, clinical utility, and model transparency, laying a foundation for clinical translation. Fifth, rigorous standardized criteria for VPS outcome adjudication were implemented with an independent dual-physician review mechanism, reducing the risk of outcome misclassification.

### 4.5 Limitations

This study has several limitations. First, as a retrospective study, it is subject to residual confounding and selection bias. In particular, exclusion of patients undergoing VPS within 21 days may have omitted early-onset SDHC, which likely differs mechanistically from the later-onset SDHC targeted here; therefore, the present model should not be applied to early-onset disease. Second, the sample size was modest for a machine learning study (development cohort, *n* = 228; external validation cohort, *n* = 102), and the small external validation cohort (24 positive cases) resulted in wide confidence intervals for some estimates. Although nested cross-validation, a conservative feature-to-sample ratio, and external validation were used to reduce overfitting, larger multi-regional cohorts are still required. Third, despite unified VPS adjudication criteria and independent dual-physician review, VPS decisions may still have been influenced by clinician judgment and local practice patterns. In addition, both centers were located in Fujian Province, which may limit geographic and practice-level generalizability. Fourth, some patients with milder disease may have been discharged before 3 weeks, leading to non-random missingness related to disease severity; moreover, ICP data were based on intermittent lumbar puncture rather than continuous monitoring, which may have limited characterization of ICP dynamics. Fifth, several potentially informative variables were not included, including semiautomated SAH volume quantification, refined CSF biomarkers, and established IVH severity scores such as the modified Graeb score. Sixth,retrospective design and selection bias: prospective, protocol-driven multi-center cohort studies should be conducted to reduce residual confounding from unstandardized clinical decision-making. Seventh,modest sample size and geographic restriction to a single province: cross-provincial and cross-institutional external validation, ideally within a prospectively harmonized data-collection protocol, is warranted to confirm generalizability beyond Fujian Province. Eighth, ICP data derived from intermittent lumbar puncture rather than continuous monitoring: future studies should incorporate continuous invasive or non-invasive ICP monitoring to more precisely characterize high-frequency ICP dynamics that intermittent sampling may miss. Ninth,fixed day-21 landmark: flexible, multi-timepoint landmark models (e.g., updating risk estimates at days 7, 14, and 21) should be developed to accommodate the diverse EVD-weaning and discharge practices described in Section 4.3.

## 5 Conclusion

In summary, this study developed and externally validated a random forest predictive model based on multimodal dynamic data that can accurately predict the risk of SDHC after aSAH. The model integrates conventional risk factors with dynamic pathophysiological trajectories collected during hospitalization; it performed well in internal validation (AUC 0.894) and maintained stable discrimination and calibration in an independent external validation cohort (AUC 0.867). Combined with the key features identified by SHAP analysis, this model can provide valuable supplementary information for the early identification and stratified management of patients at high risk of SDHC after aSAH. Further validation in larger, multi-regional, multicenter prospective cohorts is warranted to confirm its robustness and to facilitate its translation into a clinical decision support tool.

## Data Availability

The raw data supporting the conclusions of this article will be made available by the authors, without undue reservation.
