# Supplementary material for: Development and external validation of a machine learning model based on multimodal dynamic data for predicting shunt-dependent hydrocephalus after aneurysmal subarachnoid hemorrhage: a dual-center retrospective cohort study
Source: Front Neurol. 2026 Jul 24;17:1837898. doi: 10.3389/fneur.2026.1837898 (PMC13447172; doi:10.3389/fneur.2026.1837898)
Supplement: Supplementary file 1 [file Table_1.DOC]

Supplementary Figure S1. LASSO regularization path in the development cohort.Each line represents the coefficient trajectory of one candidate feature as the regularization parameter λ increases. The vertical dashed line indicates the optimal λ selected by 10-fold cross-validation (LassoCV). Features retaining non-zero coefficients at the optimal λ were included in the subsequent ensemble feature selection procedure. The x-axis represents log(λ); the y-axis

represents the standardized regression coefficient.LASSO: least absolute shrinkage and selection operator.
